# Alcohol dehydrogenase activities and ethanol tolerance in *Anastrepha* (Diptera, Tephritidae) fruit-fly species and their hybrids

**DOI:** 10.1590/S1415-47572009005000012

**Published:** 2009-01-16

**Authors:** Eneas Carvalho, Vera Nisaka Solferini, Sergio Russo Matioli

**Affiliations:** 1 Laboratório de Parasitologia, Instituto Butantan, São Paulo, SPBrazil; 2 Departamento de Genética e Biologia Evolutiva, Instituto de Biociências, Universidade de São Paulo, São Paulo, SPBrazil; 3 Departamento de Genética e Evolução, Instituto de Biologia, Universidade Estadual de Campinas, Campinas, SPBrazil

**Keywords:** alcohol dehydrogenase, *Anastrepha*, hybrids, ethanol tolerance, Tephritidae

## Abstract

The ADH (alcohol dehydrogenase) system is one of the earliest known models of molecular evolution, and is still the most studied in *Drosophila*. Herein, we studied this model in the genus *Anastrepha* (Diptera, Tephritidae). Due to the remarkable advantages it presents, it is possible to cross species with different *Adh* genotypes and with different phenotype traits related to ethanol tolerance. The two species studied here each have a different number of *Adh* gene copies, whereby crosses generate polymorphisms in gene number and in composition of the genetic background. We measured certain traits related to ethanol metabolism and tolerance. ADH specific enzyme activity presented gene by environment interactions, and the larval protein content showed an additive pattern of inheritance, whilst ADH enzyme activity per larva presented a complex behavior that may be explained by epistatic effects. Regression models suggest that there are heritable factors acting on ethanol tolerance, which may be related to enzymatic activity of the ADHs and to larval mass, although a pronounced environmental effect on ethanol tolerance was also observed. By using these data, we speculated on the mechanisms of ethanol tolerance and its inheritance as well as of associated traits.

## Introduction

The alcohol dehydrogenase enzyme system (ADH) of *Drosophila* is a classical model used in understanding the question of the evolutionary relevance of enzyme polymorphism. This system permits access to several biological levels, from organismal to molecular (Chambers, 1991), and is directly related to environmental factors. As a result of this scenario, the ADH system has been one of the most studied in *Drosophila* (Chambers, 1991; Luque *et al.*, 1997; Pecsenye *et al.*, 1997). Here we used *Anastrepha* flies as models for ADH studies, owing to their remarkable ability to undergo viable inter-specific crosses between species that express different numbers of *Adh* copies and with differences in ADH related traits, a feature rarely observed in *Drosophila*.

The families Tephritidae and Drosophilidae are phylogenetically related (both belong to the Acalyptratae subsection of Schizophora, Yeates and Wiegmann, 1999), although tephritid larvae feed on fresh vegetal tissues whereas drosophilids feed mainly on fungi. Furthermore, *Anastrepha* flies are agricultural pests, remarkably jeopardizing fruit production worldwide (Aluja, 1994).

Owing to their obtaining nourishment on fruits during the larval stage, through necessity, these flies withdraw all the nutritional factors from these while ripening. Microorganisms such as yeasts attack sugary fruits and can produce high concentrations of metabolites (Janzen, 1977). One of the most common by-products through the action of such microorganisms is ethanol, which is toxic to flies when in high concentration (Parsons, 1983; Matioli *et al.*, 1992; Chakir *et al.*, 1993; Martel *et al.*, 1995; Pecsenye *et al.*, 1997). Accordingly, since the intake of these products is unavoidable, larvae must possess efficient detoxification mechanisms. An important element is the ADH system, which degrades 90% of the total ethanol in *Drosophila melanogaster* (Heinstra *et al.*, 1987; Geer *et al.*, 1993). ADH action *per se* is enough to convert a toxic exogenous substance (ethanol) into a common endogenous one (acetate) (Kapoun *et al.*, 1990; Chakir *et al.*, 1993; Geer *et al.*, 1993).

Several features of the ADH system in *Drosophila* are related to alcohol metabolism and tolerance (Heinstra *et al.*, 1987; Geer *et al.*, 1993). On the other hand, Geer *et al.* (1993) emphasized that many other factors may play an important role in alcohol metabolism and tolerance, such as the activity of other enzymes, the composition of the cell membrane and its susceptibility to ethanol, the intensity of signal transduction in the presence of ethanol and, finally, the physiological state of the individual larva. While ethanol tolerance is a complex trait with regard to its components, its measurement is direct and simple. What remains difficult and not totally clear is the determination of all those factors that cause the observed tolerance values.

In addition to its role as a detoxification agent, evidence from studies with *Drosophila* species indicate that the ADH enzyme is also involved in the regulation of fatty acid synthesis (Geer *et al.*, 1985; Freriksen *et al.*, 1991), and even in the use of ethanol as an energetic source, particularly at lower concentrations (Bokor and Pecsenye, 2000).

The ADH system of *Anastrepha* is similar to that of *Drosophila* in its electrophoretic patterns, the dimeric composition of the functional enzyme and the differential tissue and life stage expression of loci (Matioli *et al.*, 1986, 1992; Nascimento and Oliveira, 1997). Their ADH enzymes seem to have evolved independently although from a common ancestral gene (Ashburner, 1998). Brogna *et al.* (2001) go as far as to suggest that ancestral genes of ADH from tephritid and drosophilid appeared earlier than the separation of these two families, prior to the Calyptratae/Acalyptratae divergence.

The number of *Adh* loci is variable in Tephritidae flies. Goulielmos *et al.* (2003) suggest that the *Adh* locus duplicated early in this family, before the emergence of various genera. Consequently, whilst some species have only one *Adh* locus (*e.g.*: *Acinia fucata* and *Rachiptera limbata*), many others have two (*e.g.*: *Bactrocera oleae*, *Ceratitis capitata* and *A. fraterculus*), and some rarely observed species (*e.g.*: *A. obliqua*) even have three. Interestingly, the group with one locus lives inside inflorescences or galls, while, on the contrary, the group with two or more loci abides inside ripening fruits (Matioli *et al.*, 1992). As pointed out by Goulielmos *et al.* (2003), this observation may correlate ADH evolution with speciation through adaptation to various feeding niches. According to Eliopoulos *et al.* (2004), the isozymic-specific residues of ADH1 and ADH2 may be related to preferential binding of different alcohols or to interactions with other proteins.

We studied two species, *A. fraterculus* and *A. obliqua*, with two and three *Adh* loci, respectively (Matioli *et al.*, 1986, 1992). Intercrossing between these two species has been described (dos Santos *et al.*, 2001), and generates only hybrid females. These are fertile and can be backcrossed with males from both parental species. As a result, the parents, both hybrids and backcrosses, constitute groups with differences in both the number of *Adh* loci and their genetic background.

We considered this as an interesting model for studying the relationship between the ADH system and ethanol metabolism and tolerance. The parents, hybrids and backcrosses of *A. fraterculus* and *A. obliqua*, were studied regarding ADH activity and survival. The parameters analyzed were (1) phenotype/physiological factors (ADH activities, larval protein content and ethanol tolerance), (2) environmental factors (exposure time and ethanol concentration) and (3) genetic factors (genetic background composition).

## Materials and Methods

### Population rearing and crosses

Flies were reared from guavas, collected in infested orchards. *A.* sp 1 *nrfraterculus* was collected in Louveira, SP, Brazil, in February 1995, and *A. obliqua* in Bauru, SP, Brazil, in March 1995. Since then, the flies were being reared under laboratory conditions, with a non-fermenting artificial diet for adults and guava as a substrate for the larvae. For the crosses, the flies were separated according to sex, just after emergence. Following sexual maturity (10 days, at least), 10 virgin females of one species were placed together with 10 virgin males of the other, their number being kept constant. The cross between *A. obliqua* females and *A. fraterculus* males produced viable and fertile females. The reciprocal cross was not undertaken due to difficulties in obtaining viable offspring. The backcrosses of female hybrids with males of both parental species were also performed. Thus, we ended up with five groups to work with: *A. fraterculus*, *A. obliqua*, hybrid, backcross 1 (hybrid females backcrossed with *A. fraterculus* males) and backcross 2 (hybrid females backcrossed with *A. obliqua* males).

### Experimental design

The parental and hybrid groups have a characteristic genetic background and number of Adh gene copies: *A. obliqua* has a 100% *A. obliqua* genetic background and six copies of *Adh* genes; *A. fraterculus* has a 100% *A. fraterculus* genetic background and four copies of *Adh* genes; the hybrid has a 50% *A. fraterculus* genetic background and a 50% *A. obliqua* genetic background, and five copies of *Adh* genes, since these genes seem to have an autosomal inheritance (S. R. Matioli, unpublished data). As a consequence of the recombination and chromosomal segregation in hybrid meiosis, the *Adh* gene copies and the genetic background of the backcrosses cannot be precisely deduced. However, as a group and on an average, backcross 1 had a 75% *A. fraterculus* genetic background and a 25% *A. obliqua* one, whereas backcross 2 had the reverse, a 25% *A. fraterculus* genetic background and a 75% *A. obliqua* one. The average *Adh* gene copies in the backcrosses cannot even be estimated, since these genes were not detected in the genome and its segregation is not as yet understood.

### Ethanol exposure

Third instar larvae were exposed to ethanol. The exposure was carried out in Petri dishes sealed with PVC film, each with a cellulose sponge soaked in a solution containing ethanol at different concentrations, 0.15 M NaCl (to maintain the osmotic equilibrium) and 1% glucose (to minimize the use of ethanol as a source of energy or carbon). This experiment was carried out at 25 °C in the absence of light. In each exposure experiment and after the first 12 h of exposure, the larvae were transferred to new Petri dishes with fresh solutions at the same ethanol concentration.

Two protocols of exposure to ethanol were employed:

(1) *Exposure to 8% ethanol* for 28 h. One hundred larvae of each group were treated and then frozen in liquid nitrogen.

(2) *Exposure to 0%, 8%, 12%, 16% and 20% ethanol.* One hundred larvae per group were exposed to the five concentrations, twenty to each. They were examined every four hours over a period of 28 h, whereupon immobilized and stretched larvae were considered as dead. These were then removed and frozen in liquid nitrogen, for later measurement of ADH activities and protein contents. The remaining larvae, whether dead or alive, were frozen. Contracted specimens or those in the pupal stage were considered as alive.

### Lethal concentration 50 (LC 50) determination

The concentration required to kill half of the larvae exposed during a given time was called the Lethal Concentration 50 (LC 50), and was calculated for all exposure-times (4, 8, 12, 16, 20, 24 and 28 h) for those exposed, according to protocol 2.

LC 50 was calculated by using “EPA PROBIT ANALYSIS” software, from the Ecological Monitoring Research Division – Environmental Monitoring Systems Laboratory – U. S. Environmental Protection Agency – Cincinnati, Ohio 45268, available in their website. When either the model requirements or the heterogeneity test (from EPA software) based on the Chi-square distribution were not satisfied, the calculation was either not carried out, or if so, did not have a measurable error.

### Specific enzymatic activity and determination of enzymatic activity per larva

ADH enzymatic activity was determined for the exposed larvae of both protocols 1 and 2. We measured the specific enzymatic activity, which is the enzymatic activity of ADH per protein content (unit: μMol NADH x min^-1^ x mg total protein^-1^), as well as the enzymatic activity per larva (unit: μMol NADH x min^-1^), which is the enzymatic activity of ADH for each individual.

The larvae were removed from the liquid nitrogen and immediately ground up in 50 μL of a pre-cooled homogenization buffer (0.15 M Tris-HCl pH 8.5, EDTA 1 mM, 0.05% β-mercaptoethanol), to be then kept on ice. After homogenization, the samples were centrifuged at RCF = 20800 g for 20 min, and maintained at 4°. Ten microliters of the aqueous phase were mixed with one milliliter of the reacting solution (30 mM isopropanol and 3 mM NAD+ in a 0.15 M Tris-HCl pH 8.5 buffer) pre-heated to 30 °C. NADH formation in this solution was determined every 15 s during a period of 5 min, through spectrophotometry at 340 nm. The temperature was kept at 30 °C and enzymatic activities were calculated from data collected in the first 165 s, so as to avoid substrate limitation. ADH activity was estimated by linear regression. In order to reach the *Vmax* of ADH, the concentrations of isopropanol and NAD+ in the reacting solution were at least ten times higher than the *Km* calculated for *A. fraterculus* ADH (S. R. Matioli, unpublished data).

### Protein content

Protein content was determined by using the Bradford (1976) method.

### Effects of the developmental environment on alcohol tolerance

To verify the effects of the developmental environment on ethanol tolerance, we reared larvae of both parental species on guava, mango or papaya, the fruits being placed in cages with adult populations. Three distinct samples (20 larvae each) of *A. fraterculus* and two distinct samples (20 larvae each) of *A. obliqua* were used. Two samples of *A. fraterculus* and one of *A. obliqua* were reared on guava, one of *A. fraterculus* on papaya and one of *A. obliqua* on mango. Third instar larvae were collected from the decaying fruits and exposed to ethanol, as previously described in protocol 2. The mortality-data thus obtained was used to calculate LC50.

### Statistical analysis

*General procedures*: Statistical analyses were carried out with JMP software (SAS Institute Inc., Release 5.1.2). Enzymatic activity data were transformed into natural logs, so as to assure normal distribution. For larvae exposed to ethanol according to protocol 1 (no environmental variation), we carried out ANOVA for comparison of sample means, and Student's *t* test as well as the Tukey-Kramer HSD test for pair-wise comparison of means. In all statistical tests, we considered the significance level at 0.05.

Groups of dead and live larvae were also compared to validate measurements (enzymatic activities and protein content) carried out on dead larva, as well as to verify whether there was any detectable ADH or protein degradation that could take place in the period of four hours after death. This was carried out by comparison of means (*t* test) with the residues saved after multiple regression analysis, in order to eliminate effects of other variables.

*Simple regression when environmental conditions were constant-Exploring the inheritance of the traits:* Larvae submitted to protocol 1 were exposed to the same ethanol concentration (8%) over a constant time (28 h), whereby environmental conditions were maintained fixed. Thus, any variation observed in enzymatic activities and protein content could be analyzed only in terms of the average composition of the genetic background. We carried out simple regressions (linear and polynomial) of genetic variation against (1) protein content (2) specific enzymatic activity and (3) enzymatic activity per larva. The best-fit-curve among the different degrees was chosen according to its F value.

*Multiple regression when all variables were varying-Estimating the level of the effect of each variable on traits:* When all the larvae exposed in protocols 1 and 2 were analyzed together, there were variations in environmental (time of exposure and ethanol concentration), genetic (composition of the genetic background) and phenotype/physiological (enzymatic activities and protein content) factors. To model some of these variables in terms of the remainder, we performed multiple regressions so as to discover the role played by each of these variables in the determination of that variable of interest. The modeled variables were specific enzymatic activity and time of resistance to ethanol (time elapsed until death). Nevertheless, in the latter case (multiple regression for time of resistance to ethanol), only data from larvae exposed to protocol 2 and that were considered as dead, were utilized. The relative importance of each regressor in affecting the modeled variable was inferred by its standardized partial angular coefficient, this being the angular coefficient found for each regressor multiplied by the ratio of its standard deviation and divided by the standard deviation of the modeled variable (Zar, 1999).

In order to choose the independent variables employed in each model, we used a factorial combination among all possible variables. Following this, we used a stepwise selection in these regressors, to keep the most informative ones. Stepwise regression was performed in the backward direction, regarding variable hierarchy, for the presence of significant composite variables, the variables that compose it cannot be withdrawn from the model, even though they are non-significant.

## Results

### General procedures

For all the variables analyzed – data from protocol 1 - the ANOVA test was significant (p < 0.001), which implies that sample means were significantly different from one another. Thus we analyzed them by pair-wise comparison for a more detailed view (data not shown).

### The use of dead larvae is plausible

In protocol 2, we described a new methodology of ethanol exposure in which larvae are collected after death. This new methodology allows for directly co-relating data on individual phenotypes and physiological state up to the time of death, which is not possible otherwise, thereby making this a highly potential process. Thus, we were able to create a model of mortality regarding variables that were measured in the individual larva. For validation of the use of dead larvae, we compared the mean values (with a *t* test) for all measured variables (specific enzymatic activity, enzymatic activity per larva and protein content) between the groups of both dead and live larvae. To eliminate the effects of environmental and genetic variation, we built a regression model (p < 0.001) and saved the residual values before mean comparison. The result was that both groups were statistically indistinguishable for all measured variables, hence validating the use of dead larvae (for the *t* test, p = 0.66 for protein content, p = 0.87 for enzymatic activity per larva and p = 0.58 for specific enzymatic activity).

### Lethal concentration 50 (LC 50) data shows apparent heterosis

**Figure 1:**
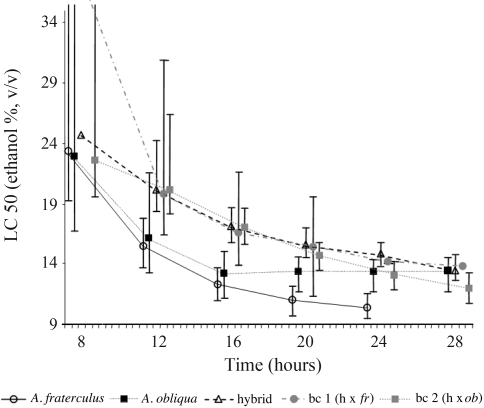
Lethal concentration 50 (LC 50) of each population (in percent ethanol), on the Y-axis, and exposure times (in hours) on the X-axis. Points in the same time-class were slightly displaced to assure adequate visualization. Limits of confidence interval are given (p = 0.05) for each LC 50 value, the absence of overlap allowing for statistical comparison. The limits of confidence and LC 50 could not be calculated for all datasets. Legends: bc1(hx*fra*): backcross of hybrid and *A. fraterculus*; bc2(hx*obl*): backcross of hybrid and *A. obliqua.*

Figure 1 profers a summary of the results for LC 50. In most cases, the 95% confidence interval overlapped, thus the greater part of LC50 values could not be confidently distinguished. Even so, backcrosses appeared to be more tolerant than parental. *A. fraterculus* also presented many significantly lower values than those of the other groups. Thus the *A. fraterculus* sample showed significantly less tolerance to ethanol than the remainder.

### Exploring the inheritance of the traits – Simple regressions when environmental conditions were constant

All the regressions obtained were significant (p < 0.001). For (a) - protein content – we obtained a first degree function, with a positive slope, for (b) - specific enzymatic activity – we also obtained a first degree function with a negative slope, and for (c) - enzymatic activity per larva – we obtained a third degree polynomial. First degree functions are characteristic of additively inherited traits, while a third degree polynomial is not clearly related to any particular inheritance pattern.

**Figure 2:**
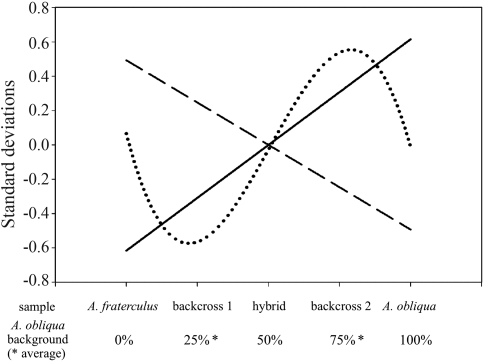
Pattern for each analyzed variable as a function of equivalent sets of values in the X-axis. Variables plots: protein content (line); specific enzymatic activity (dashed); enzymatic activity per larva (dotted). The X-axis is represented by samples and their related *A. obliqua* genetic background composition values (in percentages). Each variable was normalized by its standard deviation, thus the Y-axis represents the standard deviations for all the variables.

We plotted these results on a single graph (Figure 2), through standardizing the magnitude of each variable by subtracting the mean for each value and dividing it by the standard deviation. Thus, the Y axis presents the variation of the variables in standard deviations.

### Estimating the level of effect of each variable on traits – Multiple regressions when all variables were varied

*Specific enzymatic activity*: We carried out a multiple regression for specific enzymatic activity as the dependent variable. The independent variables were time of ethanol exposure, ethanol concentration, composition of average genetic background and a factorial combination of all these variables. Table 1 shows the fit of the model, its variance analysis and the relative effect of each variable on specific enzymatic activity.

*Survival time*: The time elapsed until death may be considered as a measure of ethanol tolerance. Based on this, we used survival time as a dependent variable. As independent variables, we used ethanol concentration, protein content, specific enzymatic activity, genetic factors, composition of the average genetic background and a factorial combination of all. Table 2 shows the fit of the model, its variance analysis and the relative effect of each variable on survival time.

### Effects of the developmental environment on alcohol tolerance

**Figure 3:**
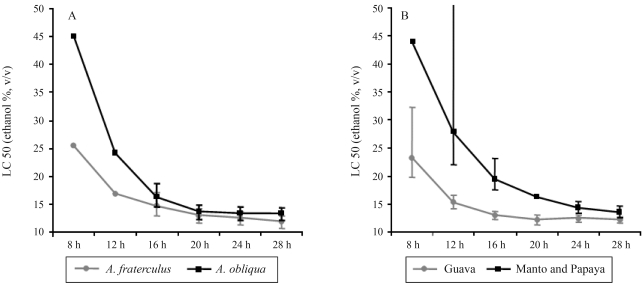
A. LC 50 (ethanol concentration in percent – Y-axis) according to exposure time (in hours – X-axis). Larvae exposed to ethanol were grouped according to species. The limits of confidence interval (p = 0.05) are given for each LC 50 value, the absence of overlap allowing for statistical comparison. The limits of confidence could not be calculated for all datasets. B. LC 50 (ethanol concentration in percent – Y-axis) according to exposure time (in hours – X-axis). The larvae exposed to ethanol were grouped according to the rearing fruit. The respective limits of confidence interval (p = 0.05) are given for each LC 50 value, the absence of overlap allowing for statistical comparison. The limits of confidence could not be calculated for all datasets.

The species *A. obliqua* (one group reared on guava and another on mango) was the most tolerant to ethanol, whereas *A. fraterculus* (two groups reared on guava and one on papaya) was the most sensitive, although with only a very slight difference (Figure 3A). However, when larvae reared on guava (two groups of *A. fraterculus* and one of *A. obliqua*) were analyzed together as a single group, and the larvae reared on papaya and mango (one group each of *A. fraterculus* and *A. obliqua*) were also analyzed together as another separate group, we observed a significant difference in ethanol tolerance between these groups (Figure 3B), the guava being more sensitive and both the papaya and mango more tolerant.

## Discussion

The methodology applied in exposure protocol 1 minimizes the effects of environmental variation, so that sample-response can be mostly attributed to genetic effects. Under these conditions, we detected an additive inheritance pattern in both protein content and specific enzymatic activity (Figure 2). Protein content in the samples was directly proportional to the genetic background of *A. obliqua*. On the other hand, specific enzymatic activity was inversely related to the genetic background of *A. obliqua*, signifying less enzymatic activity per tissue as the genetic background of this species increases. Similarly, dos Santos *et al.* (2001) also reported several intermediate phenotypes between hybrids of *A. fraterculus* and *A. obliqua*, as expected for additively inherited traits. However, we obtained different results for specific enzymatic activity when we analyzed data from protocols 1 and 2 together. Based on this finding, it may be suggested that specific enzymatic activity is a more complex inheritable trait (more detailed discussion below).

On the other hand, enzymatic activity per larva could not be fitted into either the additive or dominant models of inheritance (Figure 2). Its pattern could be best explained as the result of epistatic effects. Epistasis seems to be almost universally found in complex genetic systems as well as in apparently simple Mendelian traits (Matioli and Templeton, 1999; Templeton, 2000). Moreover, it has been shown that the *Drosophila* ADH system is subject to the influence of several epistatic effects (McKechnie and Geer, 1998; Pecsenye and Saura, 1998; Leal and Barbancho, 1992; Laurie and Stam, 1994; Stam and Laurie, 1996). *A. fraterculus*, *A. obliqua* and the hybrid disclosed a similar medium value for enzymatic activity. However, backcrossing of the hybrid with *A. fraterculus* resulted in a decrease in enzymatic activity whereas backcrossing with *A. obliqua* resulted in an increase. Therefore, the genetic background of the parental species is the factor responsible for enhancing or reducting the enzymatic activity (Figure 2). Both effects can be observed in hybrids. However, according to current evidence, hybrid inferiority is more frequent, while hybrid superiority (heterosis) is rarer (Burke and Arnold, 2001). We also observed a positive correlation (p < 0.001) between protein content and enzymatic activity per larva for all the samples, except for backcross 2 (data not shown). The size of those organs in which ADH is expressed may be a determinant of ADH expression level, or the control of ADH expression may even be influenced by the same factors that regulate determination of the size of developing larvae. As such, and independent of the causes of such enzymatic activity-size association, its breakdown could be related to the heterosis observed in backcross 2.

When comparing our *Anastrepha* data with published data on *D. melanogaster*, broadly speaking, it seems that the specific enzymatic activity (Stam and Laurie, 1996) and ethanol tolerance (Chakir *et al.*, 1996) of *D. melanogaster* are higher than the ones observed in *Anastrepha*. This may be attributable to different evolutionary pathways followed by the two. *Drosophila* is a saprophytic organism, feeding on the micro-flora that develops on senescent fruits (Parsons and Stanley, 1981), whereas *Anastrepha* larvae feed on fruits at an earlier stage, from the period of unripe fruits up to the beginning of the decomposition process (Zucoloto, 2000). Once ethanol concentration increases at the time of ripening, on an average, *Drosophila* will live in environments with a higher concentration than *Anastrepha* during its life cycle. Thus, these different environmental conditions can have lead to the different adaptations in each genus.

The effects-model (Table 1) indicates that specific enzymatic activity is increased by a longer ethanol exposure time and concentration. This may reflect the induction of *Adh*, similar to that reported by several authors regarding the same process in *Drosophila* (Kapoun *et al.*, 1990; Martel *et al.*, 1995; Pecsenye *et al.*, 1997; Pecsenye and Saura, 1998).

The effects-model also suggests that specific enzymatic activity increases with the *A. obliqua* genetic background (p < 0.0001 - Table 1). Hence, we observed two opposite effects for the *A. obliqua* genetic background in specific enzymatic activity, for in a simple regression (Figure 2), there was a negative effect, whereas in a multiple regression (Table 1), the effect was positive. As in the simple regression data were only obtained in the scant conditions of 8% ethanol, it appears that under these conditions, a more ample *A. fraterculus* background leads to higher efficiency in ethanol degradation. On the other hand, for multiple regression analyses data on larvae exposed to ethanol concentrations higher or equal to 8% was used. Under these conditions, the more ample the *A. obliqua* background, the higher the efficiency in ethanol degradation. We can hypothesize that *A. fraterculus* is more efficient in using ethanol as an energy resource (at lower concentrations, above 8%) and *A. obliqua* was more efficient in degrading ethanol to avoid toxic effects, which is in agreement with the data from LC 50 and the regression model for time of survival. Both sets of data analysis suggest that *A. obliqua* is more resistant to ethanol than *A. fraterculus*.

The survival of larvae exposed to ethanol (whose toxicity was placed in evidence by our model) was dependent on several factors. Regarding the effects of the genetic background, data from LC analysis (Figure 1) suggest that there was hybrid superiority, thus characterizing a heterosis effect, although there was no clear statistical significance for this statement. The multiple regression model for time of survival, both in greater detail and with statistical significance (p < 0,001), points to the *A. obliqua* genetic background as being the most important variable for larval survival (Table 2). This could not be detected in LC analysis. Nevertheless, LC 50 data (Figure 1) could significantly show that the less tolerant sample is *A. fraterculus*, in agreement with the model. We also observed that protein content was the second most important variable in the increase in survival-time. The third most relevant variable was specific enzymatic activity. Thus, since the increase in the *A. obliqua* genetic background increases the value of these two variables (Figure 1 and Table 1), we can say that the *A. obliqua* genetic background is decisive to enhancing larval survival in the presence of high ethanol concentrations. As can be seen, ethanol tolerance is a very complex trait, which is not explained only by ADH activity, although ADH is necessary in the overall model.

Notwithstanding, environmental factors seem to be the key to ethanol resistance. Data from diverse *Anastrepha* species reared on different fruits showed that ethanol resistance is more related to the fruit in which the larva has been reared than to the population itself and even more so than the species (Figure 3). When larvae were grouped according to the fruit, there were greater differences in ethanol tolerance than when grouped according to species. Larvae reared on papayas or mangoes were more tolerant than those reared on guavas. Geer *et al.* (1993) pointed out that diet can influence stress-tolerance, and that levels of vitamins or nutrients can affect tolerance under alcoholic stress. If we consider fruit as a complex environment, it is difficult to say what affects ethanol tolerance. Larger fruits such as mango and papaya, can, however, provide the larval population infesting it with higher quantities of nutrients than smaller ones such as guava, which could result in an increase in larval mass. Since protein content seems to be one of the most important factors in ethanol tolerance, then larger fruits may indirectly influence larvae to be more tolerant to ethanol than smaller ones. This may explain our data.

We hypothesize that the enzymatic activity of larvae exposed to ethanol can reach a physiological maximum. The concentration used here (≥ 8%) was much higher than that normally observed in fruits infested by *Anastrepha* (~ < 1%, Matioli *et al.*, 1992). Consequently, larvae were kept in an extreme situation and could reach their maximum ADH enzymatic activity, in which all available ADH enzymes were fully dehydrogenating ethanol. In this situation, the maximum potential of the ADH system in helping to avoid ethanol toxicity could be reached, and subsequent increases in ethanol concentration would lead to the more preeminent effect of protein content in enhancing survival. As a point of discussion, under conditions of lower ethanol concentrations and longer exposure periods (closer to the natural environmental conditions of larvae), and when ethanol is predominantly used as an energy source, specific enzymatic activity would have greater importance in larval survival. A high concentration was used here since there was almost no mortality with lower ones. In *Drosophila* and in concentrations lower than 7.5%, ethanol is used as an energy source with no toxic effects (Sanches-Canete *et al.*, 1986). The same could occur with *Anastrepha*. Results from Bokor and Pecsenye (2000) indicate that ADH are important in ethanol utilization when used as nutrients, but when ethanol concentration becomes toxic, survival (as related to ethanol tolerance) is not associated with the *Adh* genotypes, but to other unknown genetic factors. In our case, if the ethanol metabolism of *Anastrepha* is similar to *Drosophila*, a candidate factor for this other variable could be the protein content of larvae, an indicative of body mass. Ethanol tolerance seems to be mainly mediated by the capacity to metabolize the product, decrease its concentration in hemolymph and thus protect the nervous system (David, 1988). Selective pressure for an increase in body mass can lead to an increase in the amount of ethanol that can be ingested before reaching toxic internal concentration in hemolymph, thus theoretically allowing for an increase in ethanol consumption.

Through this rare and informative model, in which the crossing of species with differences in genetic constitution, as in phenotypic traits, is made possible, we demonstrated that there are genetic factors acting on the enzymatic activity of ADH and on ethanol tolerance as well, which also seem to be largely affected by environmental conditions. Furthermore, we suggest the mechanisms involved in the determination of these traits.

## Figures and Tables

**Table 1:** Multiple regression for specific enzymatic activities.

A – Summary of fit			
Number of sampled larvae	Coefficient of determination (R^2^)		Mean of response

997	0.1609		7.1743

B – Analysis of variance			
Source of variation	Degrees of freedom	F ratio	Prob > F

Model	5	38.0017	0.0001
Error	911		
Total	996		

C – Regressors^1^			
Regressor	Standard slope	F ratio	Prob > F

Background (*A.obliqua*) *Time of exposure	-1.65	28.3086	0.0000
Time of exposure	1.07	14.8684	0.0001
Background (*A.obliqua*)	0.90	17.8292	0.0000
Background (*A.obliqua*) *Ethanol concentration	-0.83	7.1257	0.0077
Ethanol concentration	0.76	6.3310	0.0120

^1^The cross between variables is indicated by an asterisk. Regressors were placed in order according to the absolute value of their standard slope. The latter indicates the level of regressor effect on the modeled variable; positive standard slopes signify an increasing effect in specific enzymatic activity, whereas negative standard slopes signify a decreasing effect.

**Table 2:** Multiple regression for survival time from larvae exposed to ethanol.

A – Summary of fit			
Number of sampled larvae	Coefficient of determination (R^2^)		Mean of response

243	0.2783		15.5884

B – Analysis of variance			
Source of variation	Degrees of freedom	F ratio	Prob > F

Model	6	15.1666	0.0001
Error	236		
Total	242		

C – Regressors^1^			
Regressor	Standard slope	F ratio	Prob > F
Background (*A.obliqua*) *Specific ADH activity	-5.10	8.7895	0.0033
Background (*A.obliqua*)	4.90	9.6111	0.0022
Background (*A.obliqua*) *Protein content	-2.36	12.8113	0.0004
Protein content	2.17	12.0660	0.0006
Specific ADH activity	1.57	5.6138	0.0186
Ethanol concentration	-0.29	24.1479	0.0000

^1^The cross between variables is indicated by an asterisk. Regressors were placed in order according to the absolute value of their standard slope. The latter indicates the level of regressor effect on the modeled variable; positive standard slopes signify an increasing effect in survival time, whereas negative standard slopes signify a decreasing effect.
